# Biologically plausible local synaptic learning rules robustly implement deep supervised learning

**DOI:** 10.3389/fnins.2023.1160899

**Published:** 2023-10-11

**Authors:** Masataka Konishi, Kei M. Igarashi, Keiji Miura

**Affiliations:** ^1^Department of Biosciences, School of Biological and Environmental Sciences, Kwansei Gakuin University, Sanda, Hyogo, Japan; ^2^Department of Anatomy and Neurobiology, School of Medicine, University of California, Irvine, Irvine, CA, United States

**Keywords:** backpropagation, feedback alignment, deep learning, neuromorphic engineering, entorhinal cortex, dopaminergic neurons, olfactory system, biological plausibility

## Abstract

In deep neural networks, representational learning in the middle layer is essential for achieving efficient learning. However, the currently prevailing backpropagation learning rules (BP) are not necessarily biologically plausible and cannot be implemented in the brain in their current form. Therefore, to elucidate the learning rules used by the brain, it is critical to establish biologically plausible learning rules for practical memory tasks. For example, learning rules that result in a learning performance worse than that of animals observed in experimental studies may not be computations used in real brains and should be ruled out. Using numerical simulations, we developed biologically plausible learning rules to solve a task that replicates a laboratory experiment where mice learned to predict the correct reward amount. Although the extreme learning machine (ELM) and weight perturbation (WP) learning rules performed worse than the mice, the feedback alignment (FA) rule achieved a performance equal to that of BP. To obtain a more biologically plausible model, we developed a variant of FA, FA_Ex-100%, which implements direct dopamine inputs that provide error signals locally in the layer of focus, as found in the mouse entorhinal cortex. The performance of FA_Ex-100% was comparable to that of conventional BP. Finally, we tested whether FA_Ex-100% was robust against rule perturbations and biologically inevitable noise. FA_Ex-100% worked even when subjected to perturbations, presumably because it could calibrate the correct prediction error (e.g., dopaminergic signals) in the next step as a teaching signal if the perturbation created a deviation. These results suggest that simplified and biologically plausible learning rules, such as FA_Ex-100%, can robustly facilitate deep supervised learning when the error signal, possibly conveyed by dopaminergic neurons, is accurate.

## Graphical Abstract

Schematic illustration of three learning rules: BP, backpropagation; FA, feedback alignment; and FA_Ex-100%, feedback alignment with 100% excitatory neurons in middle layer. BP requires the information in *W*_2_ to backprop. FA requires heterogeneity in the tentative impact of the middle layer neurons on the output. FA_Ex-100% is the most biologically plausible in the sense that it can be computed at a synaptic triad only with locally available information as explained below, but its performance is fairly good and comparable to that of BP. With the notations, $y_{1}^{i}\;:=f\left(\underset{j}{\sum }W_{1}^{ij}x^{j}\right)$, $y_{2}\;:=f\left(\underset{i}{\sum }W_{2}^{i}y_{1}^{i}\right)$, and $J:=\frac{e^{2}}{2}=\frac{{\left(y_{2}-y\;\right)}^{2}}{2}$, the gradient for BP is given by $\frac{\partial J}{\partial W_{1}^{ij}}=e\;W_{2}^{i}\;f^{{}^{\prime }}\left(\underset{j}{\sum }W_{1}^{ij}x^{j}\right)\cdot x^{j},$ whose $W_{2}^{i}$ is replaced by a random number *B*^*i*^ for FA and by 1 for FA_Ex100%. Therefore, for FA_Ex-100%, the synaptic weights in the middle layer are updated by the following rule: ${\left(\Delta W\right)}_{ij}=-0.01\frac{\partial J}{\partial W_{1}^{ij}}=-0.01\;x^{j}\;\theta \left(I_{i}\right)\;e,$ where θ is a step function and *I*_*i*_ is the current input to neuron i. This can be interpreted as (Δ*W*)_*ij*_ ∝ *pre*_*i*_ × *post*_*j*_ × *dopamine*. Interestingly, simplified and biologically plausible learning rules like FA_Ex-100% work robustly as far as the error signal, possibly conveyed by dopaminergic neurons, is accurate. 
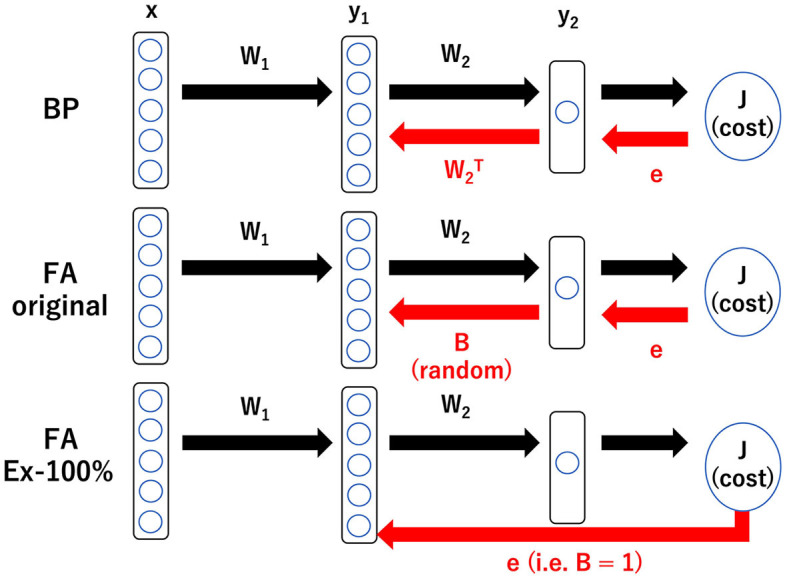

## 1. Introduction

Nowadays, deep learning with the backpropagation rule (BP) is very popular because of its high performance (Schmidhuber, 2015); accordingly, neuromorphic engineering has garnered attention (Richards et al., 2019). One of the merits of BP is that it automatically obtains an appropriate representation of features in the middle layers without manual tuning. BP efficiently leverages the explicit and repetitive function y = f(x) for neural networks to calculate gradients for updating synaptic weights. However, BP faces challenges, such as the vanishing gradient problem (Schmidhuber, 2015) and, more importantly, struggles to backpropagate across the many layers of information required for fine-tuning synaptic weights (Lillicrap et al., 2020). That is, BP is not necessarily biologically plausible because it requires sophisticated information that propagates over long distances. What synaptic learning rules are adopted by the brain?

The simplest candidate has no learning in the middle layers. An extreme learning machine (ELM) that sets the synaptic weights in the middle layers to random initial values and updates only the synaptic weights in the output layers, similar to reservoir computing, could be implemented in the brain. However, the performance of ELM is limited because it fails to fully exploit the potential of deep neural networks, as the neural representations in the middle layers do not improve during the training period.

The second well-known candidate is weight perturbation (WP), where synaptic weight changes in the middle layers are randomly sampled, similar to Markov chain Monte Carlo (MCMC) (Lillicrap et al., 2020). In this learning rule, the proposed synaptic weight changes are adopted if they reduce the error, which is conveyed as a teacher signal, possibly by the dopaminergic neurons (Schultz et al., 1997; Eshel et al., 2015, 2016; Tian et al., 2016; Watabe-Uchida et al., 2017; Kim et al., 2020; Amo et al., 2022). In other words, the gradients are not analytically computed like BP but are obtained “numerically” through trial-and-error. However, WP is inefficient as it cannot immediately identify the steepest descent direction, like BP, but rather explores better synaptic weights using random walks.

The third candidate is the recently developed feedback alignment (FA) and its variants (Lillicrap et al., 2016; Nokland, 2016; Frenkel et al., 2021). FA updates synaptic weights in the middle layers using a modified backpropagation rule, where the W_2_ term, which represents the synaptic weight vector to the output layer, is replaced with a fixed ([-1,1]-uniformly) random vector B. FA should successfully complete learning; for example, if W_2_ approaches B by the end of learning, consistent with the learning assumption (*W*_2_ = *B*). Note that the difference between BP and FA resides in the learning of synaptic weights in the middle layers; however, the learning rule for synaptic weights in the output layers remains common for both rules. Because FA is fairly heuristic, there may be room for improvement.

The candidates for the learning rule that the brain implements can be narrowed down by comparing the performances of FA and its variants to those of BP (see also Scellier and Bengio, 2017; Song et al., 2020, 2022; Meulemans et al., 2021; Millidge et al., 2022; Salvatori et al., 2022 for other potential learning rules). Learning rules that underperform the behavioral performance of mice, for example, are unlikely to be implemented in the brain.

However, most benchmarks in previous studies on FA and its variants were unsatisfactory because they used image recognition tasks. (1) There is no evidence that dopaminergic signals are used as error signals for learning in the primary and other visual cortices. (2) Specifically, there is no evidence that the “middle layers” in the visual system exhibit enough plasticity depending on the training images and their labels. (3) The BP for conventional convolutional neural networks specialized in image processing is too complex to be implemented in the brain. (4) The object recognition task requires excessively long training sequences, which do not end while the animals are alive. Given that the performance of various learning rules can heavily depend on the tasks imposed, it is very important to impose a biologically plausible task when comparing different learning rules as candidates implemented in the brain. That is, the mathematical neural network models to be constructed should cover the brain regions where the “middle layers” display enough plasticity for a given task.

Therefore, we focused on the plasticity in the entorhinal cortex (Igarashi et al., 2014, 2022; Igarashi, 2015, 2016), where dopaminergic inputs are known to exist, and constructed a mathematical model to explain it as learning in the middle layer of a deep supervised neural network. A previous study reported that during an experiment in which mice performed a task to obtain a water reward, the entorhinal cortex displayed plasticity, which can be viewed as representation learning in the middle layer, with dopamine serving as a teacher signal (Lee et al., 2021). Thus, it is worth modeling this olfactory system to elucidate learning rules in the middle layer of the brain. Furthermore, knowledge of the network structure of the olfactory system, which is evolutionarily conserved to some extent, can be utilized for mathematical modeling (Hiratani and Latham, 2022). We used a basic mathematical model of the olfactory system as a multilayer neural network, including the olfactory cortex (sensory input layer), entorhinal cortex (middle layer), and prefrontal cortex (output layer). In this study, we compared the learning performance of this model under different learning rules. A graphical summary is presented in Graphical Abstract.

## 2. Materials and methods

In this study, we performed numerical simulations in which a three-layer network solved a generalized XOR task (k-dXOR task) using various learning rules and learning parameters. All numerical calculations were implemented using handmade code in Python 3.9.13. The Python codes used to reproduce all figures are publicly available.

### 2.1. k-dXOR task

We simulated a laboratory task in which the output neuron learned the reward amount (Wang et al., 2013). As the expected reward amount is a continuous variable, we used a regression task rather than a classification task.

As the input-output function to learn, we used the k-dXOR task, where, of the d dimensions of the inputs, the first k inputs are relevant and necessary to predict the output, and the remaining d-k inputs are irrelevant. Specifically, the true input-output function to learn is assumed to be

$$\begin{array}{l} y=sign\left(x_{1}\right)sign\left(x_{2}\right)\ldots sign\left(x_{\text{k}}\right). \end{array}$$

To generate the training and test artificial data, we first randomly generated the x-coordinates (x_1_, x_2_, …, x_n_) and then determined y according to the above equation. x_i_ was generated randomly according to the normal distribution, with its expectation randomly chosen as +1 or −1 with a probability of 0.5 and standard deviation of 0.01:

$$\begin{array}{l} x_{\text{i}}={\left(random.rand\left(\right)>0.5\right)}^{*}2-1+random.randn{\left(\right)}^{*}0.01 \end{array}$$

A nonlinear task was considered because it is too easy to reflect a realistic laboratory task. Thus, we used k = 2 because it is known that rodents can perform reversal learning, which can be regarded as k = 2 (Roesch et al., 2007) and therefore, a realistic brain model should be able to solve the k-dXOR task, at least for k = 2. In the reversal learning, the emergence of the cue (or the first) stimulus upsets the entire task and reverses the output.

Learning performance was measured using the squared error of the test data or the predicted squared error. In each figure, the average and standard deviation of the predicted squared errors for 100 repeated simulations with different random seeds are plotted.

### 2.2. Three-layer neural network

Throughout the paper, we used a three-layer neural network consisting of the input layer (tentative olfactory cortex or olfactory bulb; Cury and Uchida, 2010; Miura et al., 2012; Haddad et al., 2013; Uchida et al., 2014), the middle layer (tentative entorhinal cortex; Nakazono et al., 2017, 2018; Funane et al., 2022), and the output layer (tentative prefrontal cortex; Starkweather et al., 2018). The neural activity in the input layer represents the input x of the k-dXOR task, whereas the neural activity in the output layer represents the output y. Note that we began with the olfactory representation at the olfactory cortex as an input for the neural network, although there are other early areas for olfactory information processing before the olfactory cortex, such as the olfactory bulb and olfactory receptor neurons. However, if there is low plasticity in these early areas, we believe that we can begin with a higher-level area (olfactory cortex) to simplify the model.

**Figure 1:**
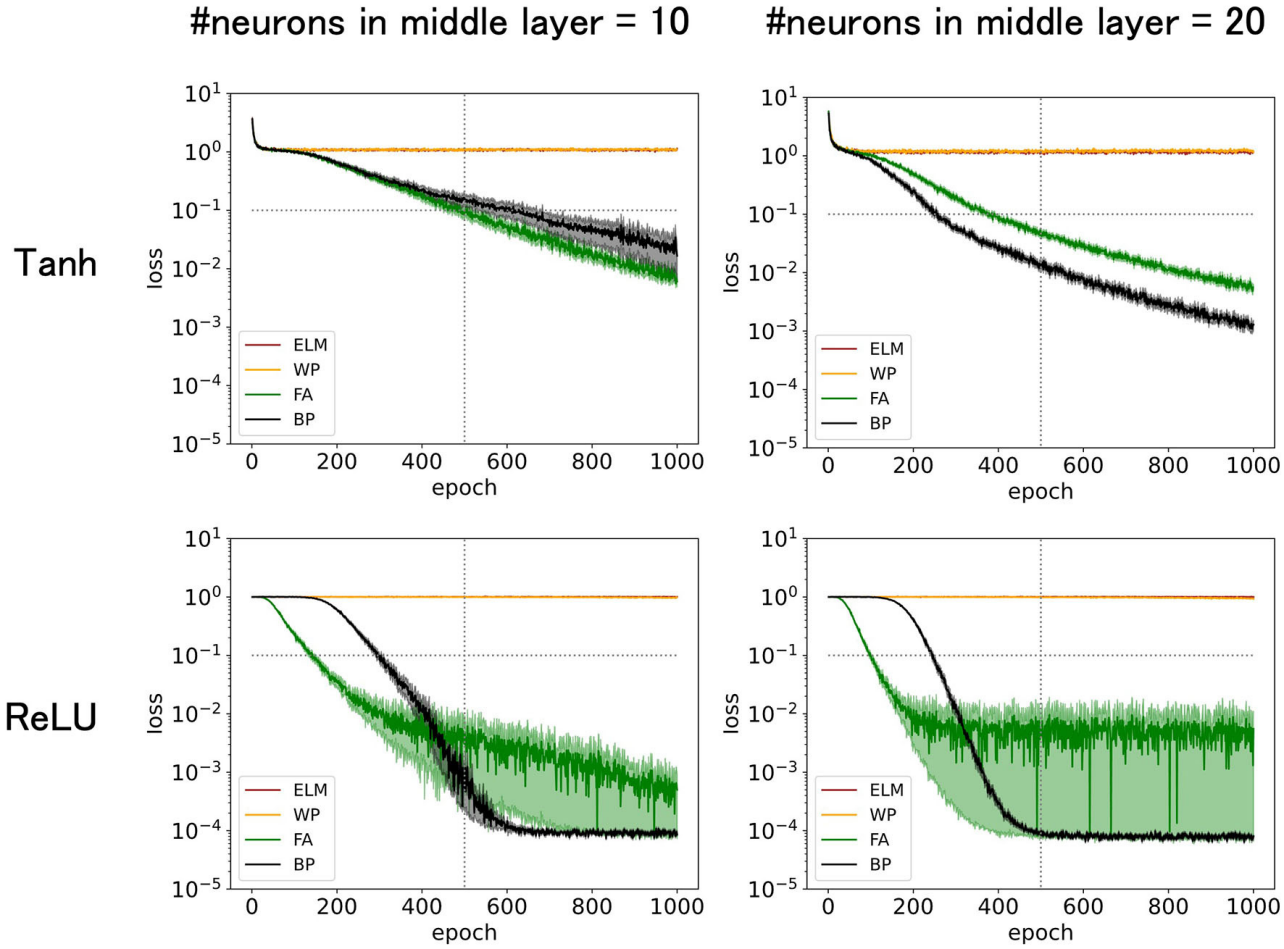
Predicted mean squared errors for four learning rules: BP, FA, WP, and ELM. The input dimension is 12, of which the relevant input dimension is two and the noise input dimension is 10. The learning rate η = 0.02 for tanh or η = 0.01 for ReLU is chosen to be large enough to maximize training speed while preserving stability. The performance of FA is comparable to that of BP. The performances increased with number of middle layer neurons and with ReLU as an activation function.

**Figure 2:**
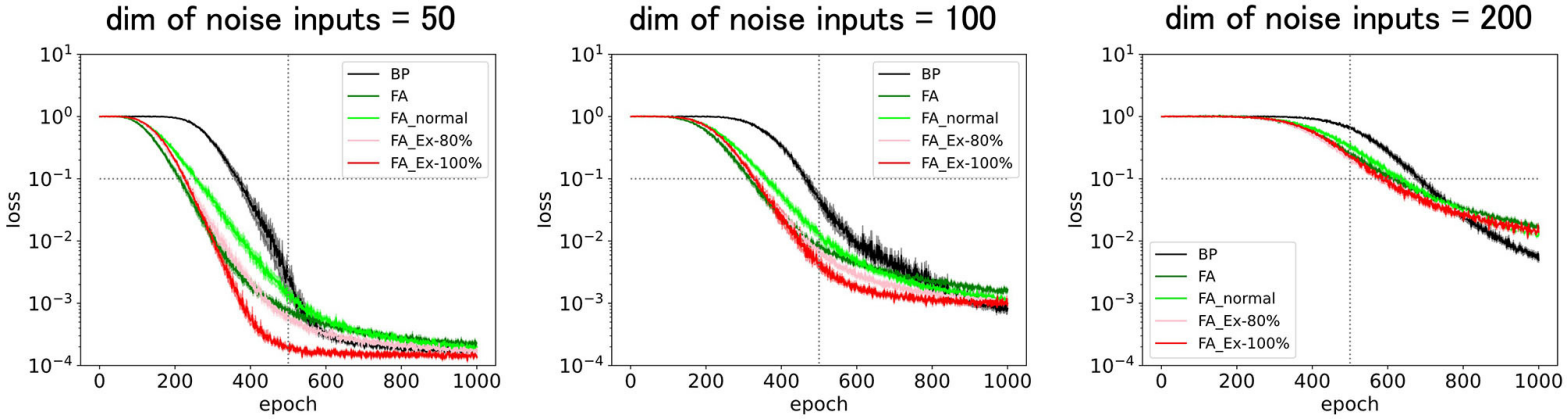
Predicted squared errors for BP, FA, FA_normal, FA_Ex-80%, and FA_Ex-100% with various noise input dimensions. The relevant input dimension is two, the number of middle layer neurons is 20, and the learning rate is η = 0.01. The performances for the variants of FA are fairly good and comparable to those of FA and BP.

**Figure 3:**
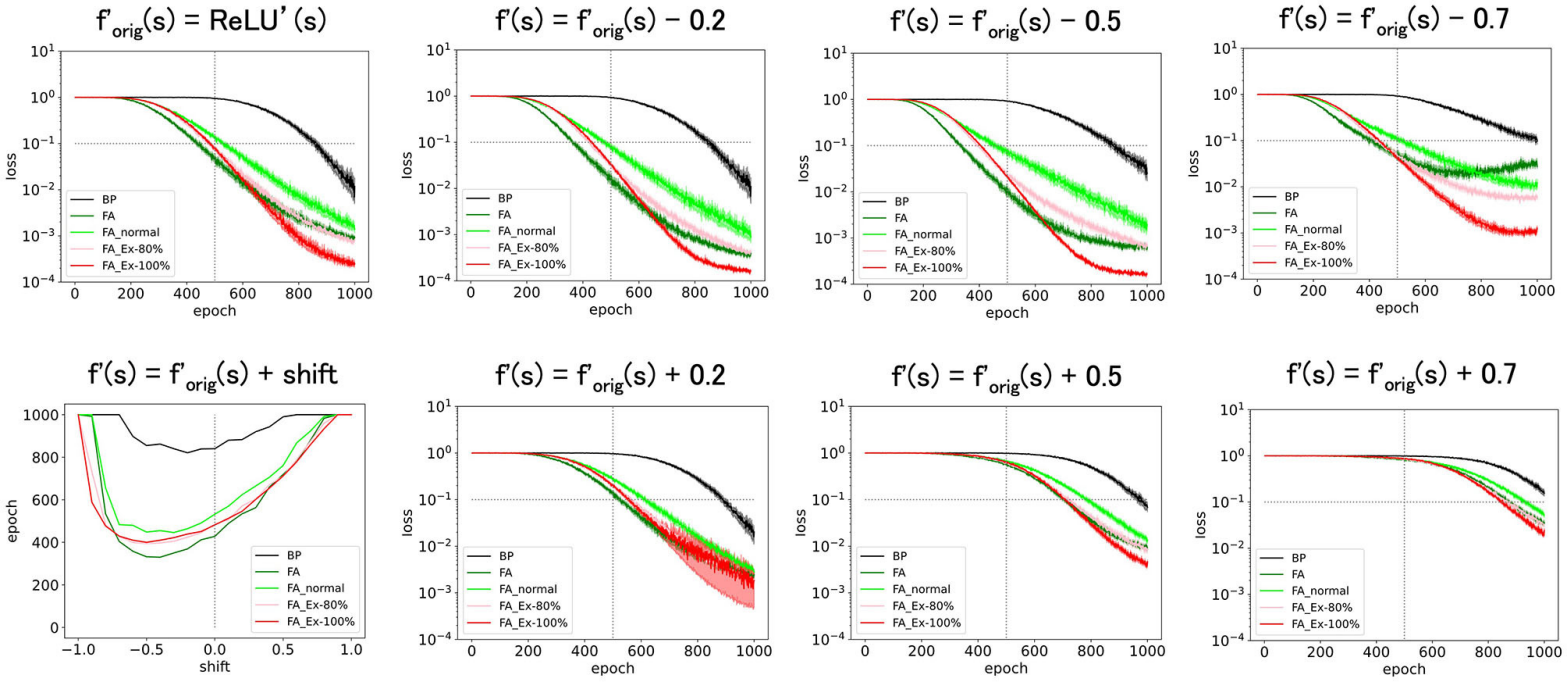
Predicted squared errors when f'(s) is shifted along y-axis. As an activation function, ReLU is used. The learning rate is 0.005 (commonly used for Figure 3 and later and the half of that for Figures 1, 2). The number of middle layer neurons is 20 **(left–bottom)**. The epoch (learning time) when the predicted squared error falls below 0.1 is plotted against the x-shift. The learning with FA or its variants is robust even if f and f' is inconsistent.

**Figure 4:**
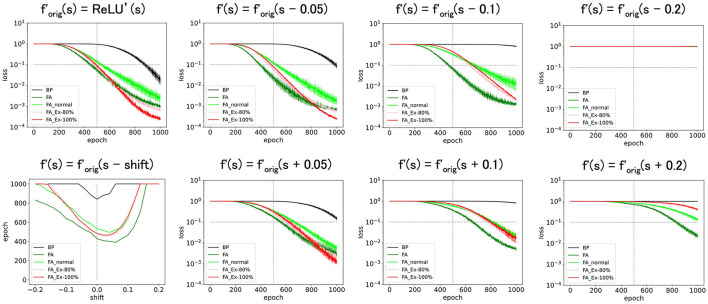
Predicted squared errors when f'(s) is shifted along x-axis. As an activation function, ReLU is used (f_orig_=ReLU). The learning rate is 0.005. The number of middle layer neurons is 20 **(left–bottom)**. The epoch (learning time) when the predicted squared error falls below 0.1 is plotted against the y-shift. The learning performance with FA or its variants is robust even if f and f' is inconsistent.

**Figure 5:**
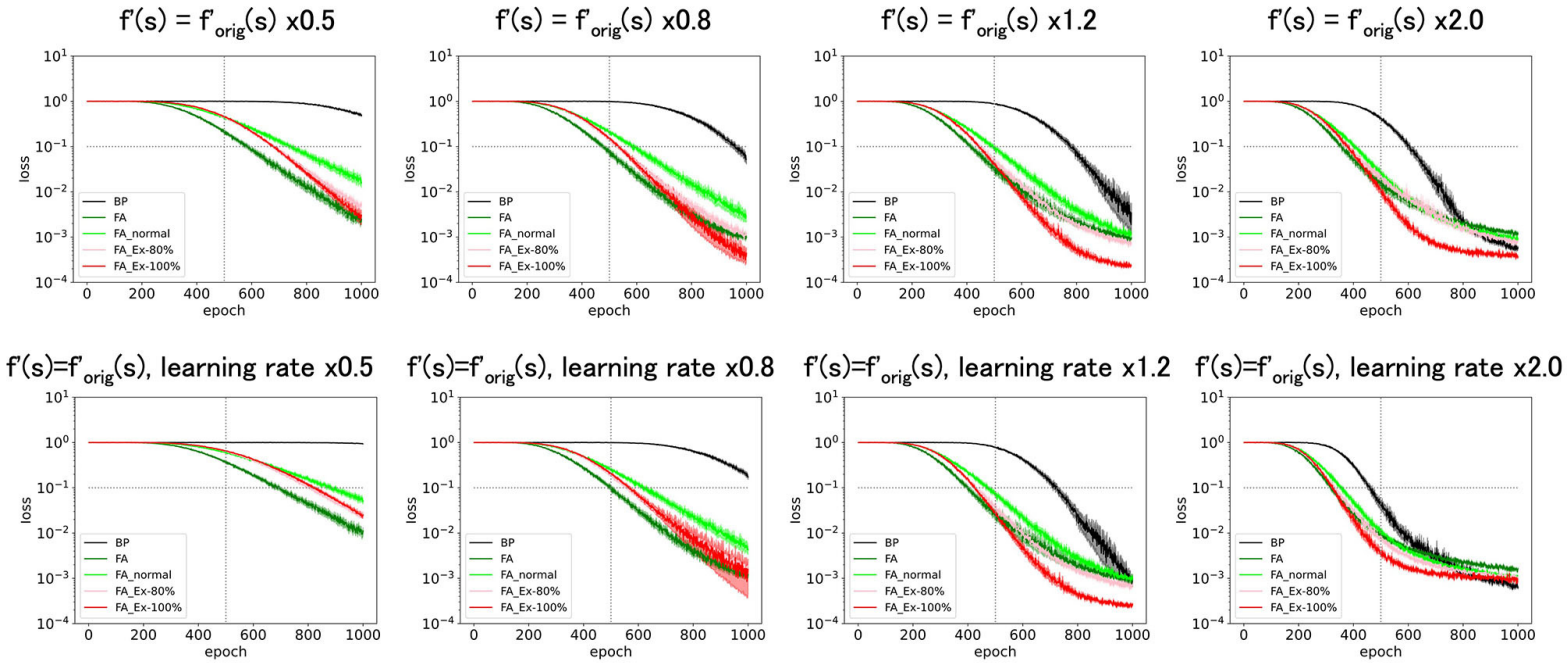
Predicted squared errors when f'(s) is scaled along y-axis. As an activation function, ReLU is used (f_orig_ = ReLU). The learning rate is 0.005. The number of middle layer neurons is 20. The learning performance with FA or its variants is robust even if f and f' is inconsistent.

**Figure 6:**
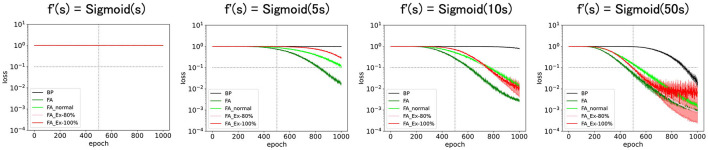
Predicted squared errors when f'(s) is scaled along x-axis as f'(s) = Sigmoid(cx). As an activation function, ReLU is used (f_orig_ = ReLU). The learning rate is 0.005. The number of middle layer neurons is 20. The learning performance with FA or its variants is robust even if f and f' is inconsistent.

**Figure 7:**
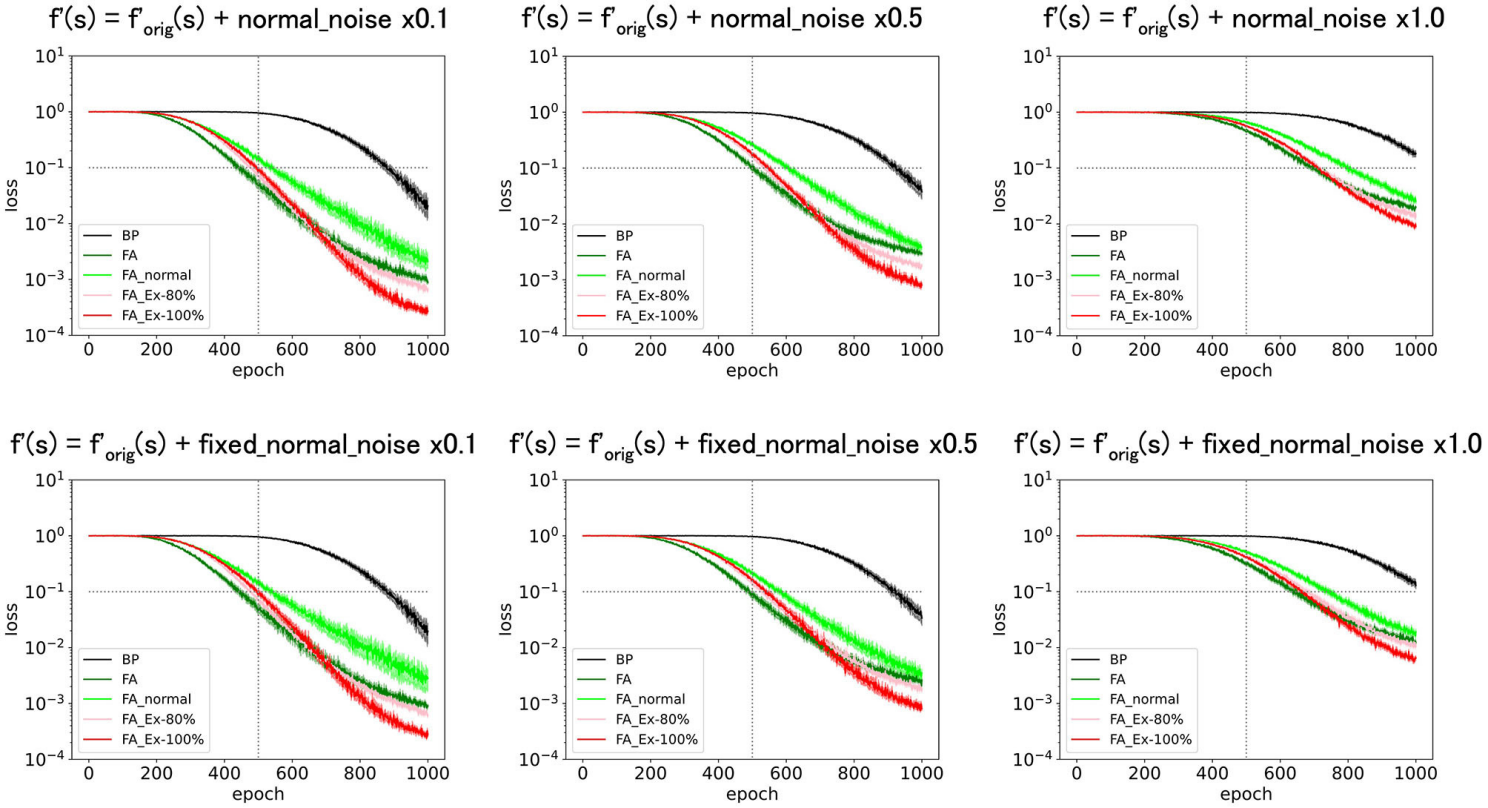
Predicted squared errors when an unfixed **(top)** or fixed normal noise **(bottom)** with various amplitudes is added to f'(s) as f'=ReLU+Noise. As an activation function, ReLU is used (f_orig_=ReLU). The learning rate is 0.005. The number of middle layer neurons is 20. The learning performance with FA or its variants is robust even if f and f' is inconsistent.

The number of neurons in the input layer is the same as that in the dimensions of the task inputs. The number of neurons in the output layer is one because the output is a scalar (one-dimensional) representing the expected amount of reward. The numbers of neurons in the middle layer were 10 or 20 for the case depicted in Figure 1 and 20 for the cases depicted in Figures 2–7.

Initial values of synaptic weights W_1_ and W_2_ were randomly chosen according to the uniform distribution [−0.01, 0.01]. Then, for training, the weights were updated using one of the following rules.

### 2.3. Learning rules

The learning rules are described as follows: Note that the difference resides only in the weight update rule for the middle layers. That is, the weight-update rule in the output layer is common for all learning rules; thus, it is the same as that for BP.

#### 2.3.1. Extreme learning machine

ELM (Huang et al., 2004, 2006) sets the synaptic weights in the middle layers to random initial values and updates only the synaptic weights in the output layers, similar to reservoir computing. In other words, the ELM never learns in the middle layers. Therefore, if the neural network does not obtain adequate representation in the layer immediately before the output layer, the task cannot be solved successfully. Specifically, a task can be solved only if the output is represented by the weighted sum of the neural activities in the layer immediately before the output layer.

#### 2.3.2. Weight perturbation

For the synapses in the middle layer, WP (Lillicrap et al., 2020) chooses a candidate for the small weight update Δ*W*_1_ randomly. Then, if the change of weights reduces the squared error for the training data, WP adopts the update and modifies the synaptic weight as *W*_1_ = *W*_1_ + Δ*W*_1_. In other words, a randomly “perturbed” weight vector Δ*W*_1_ is adopted if the perturbation decreases the cost function. To be precise, at each epoch, the elements of a candidate matrix (Δ*W*_1_)_*ij*_ are randomly proposed according to a normal distribution with a mean and standard deviation of 0 and ϵ(=0.005), respectively.

#### 2.3.3. Back propagation

BP (Richards et al., 2019; Lillicrap et al., 2020) updates the synaptic weights using the usual backpropagation rule for both the middle and output layers. The weight vector is updated according to the gradient vector to minimize the cost function (squared error). Graphical Abstract presents a concrete equation for the weight update.

#### 2.3.4. Feedback alignment

FA (Lillicrap et al., 2016) updates synaptic weights in the middle layers using the modified backpropagation rule, where the W_2_ term, which represents the synaptic weight vector to the output layer, is replaced by a fixed ([-1,1]-uniformly) random vector B. FA should finish learning successfully; for example, if W_2_ approaches B by the end of learning, consistent with the learning assumption (*W*_2_ = *B*). Graphical Abstract presents a concrete equation for the weight update. The variants of FA are described in the main text.

### 2.4. Leaning parameters

The learning rate η was set at 0.02 for tanh or 0.01 for ReLU for the case depicted in Figure 1, 0.01 for the case depicted in Figure 2, and 0.005 for the cases depicted in Figures 3–7. To simplify the comparison, we did not schedule the learning rate across the epochs. That is, we maintained a fixed learning rate within each simulation and did not change it across training epochs (time). This approach allowed us to use a learning rate that maximized performance and ensured a fair comparison of different learning rules. Note that as long as the training proceeds stably, the final performance does not essentially depend on the learning rate, except for its effect on learning speed. For example, halving the learning rate doubles the number of learning epochs required for training.

The batch size was fixed at eight. In each epoch, the cost function was measured for eight samples of training data, and a weight update was performed to reduce the cost function once per epoch. Thus, one epoch corresponds to a single weight update. Note that, as long as the total sample size for training remains the same, the batch size has minimal impact on the final performance. For example, if the batch size is reduced to four from eight, the number of epochs required to complete the learning doubles. However, the total number of samples (experimental trials) required to achieve a given level of accuracy remains unchanged. Using this trial count, one can judge whether the number of trials required is biologically realistic, which is discussed further in the Discussion section.

## 3. Results

### 3.1. Comparison of learning rules: ELM, WP, FA, and BP

In this study, using numerical simulations, we compared the performance of deep neural networks with different learning rules (ELM, WP, FA, and BP) for a task that simulated a laboratory experiment where mice predicted reward amounts (Lee et al., 2021). One important goal here is to judge the biological plausibility of the learning rules. Thus, a learning rule that underperforms in laboratory mice is unlikely to be adopted in the brain. Indeed, there is an easy and unique rule to update synaptic weights toward the output layer. Thus, the update rule in the output layer is common across different learning rules. However, the learning rule that performs best in the middle layer remains uncertain. Consequently, we compared the performance of the different synaptic update rules in the middle layers.

First, we compared the prediction performance of a three-layer neural network trained with ELM (Huang et al., 2004, 2006), WP (Lillicrap et al., 2020), BP (Richards et al., 2019; Lillicrap et al., 2020), and FA (Lillicrap et al., 2016). Although the details of each learning rule are available in the Materials and Methods section, they are briefly summarized below. ELM never updates the synaptic weights in the middle layers and maintains them at their initial randomized values. In other words, ELM only updates the synapses leading to the output layer. WP randomly proposes (small) synaptic updates in the middle layer and adopts them if they reduce the squared error for the training data in the current batch. BP updates synaptic weights using the conventional backpropagation rule for both the middle and output layers. Note that the synaptic update rule for the output layer is common to the four rules, and thus is the same as that for BP. FA updates synaptic weights in the middle layers using a modified backpropagation rule where the W_2_ term, which represents the synaptic weight vector to the output layer, is replaced by a fixed ([-1,1]-uniformly) random vector B. FA should successfully complete learning; for example, if W_2_ approaches B by the end of learning, consistent with the learning assumption (*W*_2_ = *B*).

To simulate the laboratory task where mice learned the expected amount of reward (sugar water), we trained a three-layer network consisting of the input layer (piriform cortex, *N* = 12), middle layer (entorhinal cortex, *N* = 20), and output layer (prefrontal cortex, *N* = 1) to learn the artificial data generated by the k-dXOR task, which is a generalization of XOR to various input dimensions. Further details are provided in the Materials and Methods section. Taking advantage of the fact that task difficulty can be controlled by the number of neurons in the middle layer and the input dimensions, we set the number of neurons in the middle layer to 20 and the input dimension d to 12, of which the dimension of the input that is relevant to the output k is 2 and the irrelevant dimension d_noise_ is 10. We used k = 2 entirely because rodents can perform reversal learning, which can be regarded as k = 2 (Roesch et al., 2007). Therefore, a realistic brain model should be able to solve the k-dXOR task, at least for k = 2. Note that although we use rather small noise input dimensions d_noise_, which makes the task less challenging in the real brain, the number of input or sensory neurons is relatively large. However, we believe that the number of neurons in the middle layers is also high in the real brain. Therefore, the same task can be solved by increasing both numbers in a balanced manner (Hiratani and Latham, 2022). However, owing to the limitations in computational resources, this study used a rather limited number of neurons to perform simulations, as described above. Future work may explore GPU-based simulations to increase both the input- and middle-layer neuron counts in a balanced manner. Note that as an activation function, we used either tanh (Figure 1, top), which was used in the original FA study (Lillicrap et al., 2016), or ReLU (Figure 1, bottom), which generally enhances the learning performance (Krizhevsky et al., 2017).

Figure 1 (top-left) demonstrates that the accuracy increases or the predicted squared error decreases with epochs (learning time). The performance was outstanding for conventional BP and its variant FA, where the predicted squared error dropped below 0.1, effectively solving the task of predicting sugar water amounts. FA, even in its original form, performed slightly better than BP, suggesting that biologically plausible FA may have the potential to work fairly well, particularly for specific tasks. In contrast, ELM and WP struggled to solve this problem. ELM and WP excel at simpler tasks, such as k-dXOR tasks with d_noise_ = 0 (d-k = 0, no noise input). However, as the input dimension increases and the task complexity increases, ELM and WP fall short. Given that the brain likely deals with a large number of noise inputs and solves challenging tasks, ELM and WP cannot apparently be adopted by the brain. Moreover, the training period for ELM and WP exceeds 1,000 epochs, further suggesting their implausibility in biological learning processes.

The reasons why ELM and WP underperformed may be attributed to several factors. When ELM does not have a sufficient number of neurons in the middle layers, such as in the current setting, its neural representations in the middle layer immediately before the output layer are too inadequate to solve the task. WP, which essentially randomly explores synaptic weights in the middle layers, can, in principle, eventually learn any task, but it tends to require an impractically long time to converge. This is because there are too many possibilities to explore randomly when the dimensions of the input and the space to explore are large. For example, because WP can only propose one synapse at each epoch for a possible update, it takes at least as many epochs as the number of synapses to explore all directions. Given the efficiency of exploration, BP, which skillfully utilizes the steepest descent (greedy) direction, can converge much faster, particularly for high-dimensional tasks.

Figure 1 (top-right) shows that increasing the number of neurons in the middle layer to 20 expedited the training, possibly owing to the enhanced representational capacity. In fact, the errors for FA and BP fall below 0.1 more quickly. In general, performance (generalization error) is determined by the balance between the difficulty of the task and the structure of the neural network, such as the number of neurons in the middle layer. Although the original FA uses the suboptimal weight update vector ΔW, which is not necessarily parallel to gradients like BP, the performance of FA is only slightly lower than that of BP. The time (number of training epochs) for FA to fall below 0.1 takes only 40% longer than that of BP. In fact, the performance of FA is much better than that of ELM or WP, making it a practical choice for solving the task.

Figure 1 (top-right) shows that replacing tanh with ReLU as an activation function, which is a widely recommended empirical practice, enhances performance, especially for BP and FA. ELM and WP did not show any noticeable enhancements. Notably, the predicted squared error for FA with ReLU quickly reached 0.1 in the early phase of the training. In contrast, after a considerable number of epochs, the predicted squared error for BP decreased below 0.001, faster than that for FA. However, because this asymptotic accuracy can be easily tuned by parameters, such as the scheduling of learning rates, and may be unnecessarily high for laboratory experiments, the initial phase may be more important than the asymptotic phase.

While the superiority of FA over ELM and WP in the experimental results is expected, exploring functions that depend on only a few input features is novel. Therefore, Figure 1 compares different learning rules under identical conditions, similar to a Rosetta Stone.

In the following subsection, we exclusively use ReLU as an activation function, as it demonstrates superior performance compared to tanh, as shown in Figure 1. When we used ReLU, FA demonstrated a striking performance in the early phase of training. Therefore, we continue to examine FA as a promising candidate for biological learning. ELM and WP are not considered in the subsequent figures, as they yielded relatively poor performance. Next, we attempted to further improve FA and BP by tuning various learning parameters. It is especially worth developing a variant of the FA, as the FA in its original form has already shown fairly good performance. Among the many possible variants, we wanted to explore the biologically plausible variants with adequately high performance.

### 3.2. Proposed variants of FA enhance learning performance

As shown in Figure 1, both FA and BP exhibit good performance. However, from a biological plausibility perspective, conventional BP and its variant FA, in their original forms, suffer from two challenges: (1) they require the activities of postsynaptic neurons with high accuracy, and (2) they require information that physically backpropagates across layers. Therefore, we propose new variants of FA to address these challenges. However, it is empirically known that most *ad-hoc* learning rules destabilize during training and fail. Meanwhile, learning rules based on cost functions such as BP tends to be more reliable. Thus, we base our new learning rules on BP and FA.

Fortunately, the first challenge can be resolved by simply adopting the ReLU as an activation function, which tends to outperform other activation functions. The resulting learning rule only requires ON or OFF resolutions for the activities of postsynaptic neurons and can be easily implemented in a living system with stability. This is because the differentials of the activation function for the postsynaptic neuron required to compute the learning rule are simpler for ReLU than for the tanh and sigmoid functions. For f(s) = ReLU(s), f'(s) = 0 for s < 0, or 1 for s > 0. Note that the only assumption we have proposed thus far is to use ReLU as an activation function, and no approximation to the cost function is needed to compute the differentials of the activity of postsynaptic neurons in the living system.

Note that the ReLU is not only powerful and simple in computing but also biologically plausible when rate-based models are considered. For example, it has been shown that the f-I curve (firing frequency plotted against the input current) of a realistic neuron model is well described by a ReLU (Shriki et al., 2003).

Regarding the second challenge, it is insightful to review the original FA, where the impact of the activity of a neuron in the middle layer x on the activity of a neuron in the output layer y, or dy/dx, which is used to compute the weight update Δ*W* in BP, is modified by replacing the connection matrix with output layer W_2_ with a random matrix B. Because the weight in the middle layer W_1_ is trained with this modification, learning can converge if the assumption W_2_ = B holds and everything is consistent. However, the physical substances representing B remain unclear, and information on B is required to backpropagate across the layers.

Therefore, we further modify FA slightly and use (B)_ij_ = 1 for all i and j, assuming that all middle-layer neurons have the same impact on the output layer. We call this learning rule FA_Ex-100%. However, this finding implies that only excitatory neurons exist in the middle layer. Therefore, we can further modify it to have 20% inhibitory neurons with (B)_ij_ = 1 (for i: excitatory, 80%) or −1 (for i: inhibitory, 20%) randomly according to the Bernoulli distribution. We call this learning rule FA_Ex-80%. Furthermore, we define FA_normal as the third variant of FA, where the random matrix B is neither uniform nor Bernoulli but normal. As we will demonstrate later, these three variants of FA are comparable to FA and significantly outperform ELM and WP.

Remarkably, the FA and their variants can be implemented as synaptic triads. That is, in order to compute the synaptic weight update (Δ*W*)_*ij*_, only three types of information that are available at the synapse are required: the activities of the presynaptic neuron i, the postsynaptic neurons j, and the dopaminergic neuron (error signal). Multiplying the three activities available at the synapse is a biologically plausible computation.

(1) $$\begin{array}{l} \Delta W_{1}^{ij}=pre_{i}\times post_{j}\times dopamine, \end{array}$$

The reason why only locally available information suffices to compute $\Delta W_{1}^{ij}$ is simply that *W*_2_ has been replaced by *B*. That is, if *B* = 1 (or is fixed to a constant) and the backpropagation of *W*_2_ information is no longer required, as shown in Graphical Abstract, we can simply send e (the error signal) directly to the synapses in the middle layer.

Figure 2 compares the predicted squared errors for the five learning rules, BP, FA, and the three FA variants. Here, we fixed the relevant input dimension to two and varied the noise input dimension between 50, 100, and 200 to control the task difficulty. The learning rate η = 0.01 is fixed (not scheduled), and the activation function f = ReLU and its derivative f' =ReLU' are consistently used in the equation to compute the weight update Δ*W* (this is not necessarily satisfied in the case depicted in Figures 3–7).

Figure 2 (left) shows that the performance for the variants of FA is sufficient, and, similar to FA in Figure 1, their predicted squared losses fall below 0.1 faster than that of BP. This demonstrates that learning comes into effect even if the FA does not use a uniformly random matrix B. Surprisingly, the learning rule that is implementable at a synaptic triad with only locally available information, such as FA_Ex-100% (or FA_Ex-80%), works fairly well. Furthermore, the differentials of the postsynaptic neuron's activity can easily be computed using ReLU instead of tanh as an activation function. Figure 2 (middle, right) shows that the variants of the FA can solve difficult tasks with high-dimensional noise inputs. Even a task with an input noise dimension (200) that is ten times larger than the number of middle-layer neurons (20) can be solved. Although we did not perform large-scale simulations, it is possible to solve the high-dimensional problem by balancing the input noise dimensions and the number of middle-layer neurons (Hiratani and Latham, 2022).

### 3.3. Proposed learning rules are robust and even approximated rules work

Thus far, we have proposed new learning rules as variants of the FA and have demonstrated that these variants are effective in solving challenging tasks. In FA_Ex-100% and FA_Ex-80%, the weight update Δ*W* (which is essentially a gradient vector) can be represented as a multiplication within a synaptic triad:

(2) $$\begin{array}{l} \Delta W_{1}^{ij}=pre_{i}\times f^{\prime }\left(I_{j}\right)\times dopamine. \end{array}$$

Intuitively, all we need for computing $\Delta W_{1}^{ij}$ is the impact of weight updates on postsynaptic activities, which is why *W*_2_ appeared in $\Delta W_{1}^{ij}$ for BP (see Graphical Abstract for details). Then replacing *W*_2_ by a random vector *B* is equivalent to assuming that the impact of the weight update $\Delta W_{1}^{ij}$ on the neural activity in the output layer is (proportional to) *B*^*j*^. Therefore, *post*_*j*_ in Equation 1 for FA and its variants can be expressed as $f^{\prime }\left(I_{j}\right)$, where *f* is the activation function (of the postsynaptic neuron j) and *I*_*j*_ is the current input to the j-th postsynaptic neuron. Note that because we use ReLU as an activation function *f*, *f*′ is actually a step function.

However, it may be challenging to implement Equation 2 in the brain because it requires an accurate calculation of *f*′, that is, the differential of the activity of postsynaptic neurons with respect to their input current. It is unclear whether accurate information on the activity of postsynaptic neurons can be conveyed to presynaptic neurons and if the differential of neural activity can be accurately computed in the brain. Therefore, we would like to determine whether the learning rules function even if the brain cannot accurately compute *f*′ and is forced to approximate it with some errors. Specifically, we consider a series of systematic approximations for *f*′ as variants of the learning rules and assess the extent to which they still work. We not only confirm the robustness by maintaining the accuracy but also determine if we can improve the accuracy by approximations for the heuristically derived FA and its variants, which have room for improvement. That is, although we used ReLU as the activation function *f*, *f*′ in Equation 2 for computing the weight updates is not necessarily its derivative ReLU, but something different. In this context, *f*′ is inconsistent with *f*. Specifically, to explore methods of perturbing *f*′ in a systematic manner, we consider parallel translations, scaling, and noise addition.

First, we shifted ${f^{\prime }}_{orig}$(=ReLU') along the y-axis and considered *f*′= ReLU'(s)+shift. This shift should yield a bias in computing $\Delta W_{1}^{ij}$ in **Equation 2**. However, as shown in Figure 3, these perturbed learning rules functioned fairly well as long as the shifts were sufficiently small. For example, once the weight-update rule for FA_Ex-100% in Graphical Abstract is averaged across the input x and error e for large data or long epochs, the effect of the y-shift (adding a constant) on f'(s) should approach zero when the mean of x or e is zero. This may explain why the effect of the y-shift is negligible if it is small. Similarly, the weight update rule, which can be interpreted as the multiplication of three terms, may become more stable if x, e, and f'(s) are balanced (i.e., any of the three terms have a zero-mean). In fact, strikingly, when we shifted by −0.5 along the y-axis, and *f*′ is the most balanced (zero-mean), the accuracy improved. This may provide a hint for improving accuracy, although it is uncertain whether making each term easy to cancel generally works. Note that it is generally very challenging to improve accuracy in an *ad hoc* manner, and this approach represents one of the few ways to significantly improve performance based on our experiments in this paper.

Next, we shifted ${f^{\prime }}_{orig}$(=ReLU') along the x-axis, introducing *f*′= ReLU'(s-shift). As shown in Figure 4, these perturbed learning rules functioned fairly well as long as the shifts were positive and sufficiently small. However, when the shift was negative or adequately positive, the performance deteriorated. Thus, although these learning rules are robust against x-shifts of *f*′ to some extent, it is not straightforward to significantly enhance performance solely through x-shifts.

As depicted in Figure 5 (top), amplifying f' along the y-axis as $f^{{}^{\prime }}={f^{\prime }}_{orig}$ x constant, sped up the learning. However, this result is trivial. For example, multiplying *f*′ by a constant is almost equivalent to multiplying the learning rate by the same constant (Figure 5, bottom). However, in Figure 5 (top), we do not multiply the learning constant for the output layer by the same constant, which leads to unbalanced learning. Therefore, there are discrepancies between Figure 5 (top, bottom).

Because it does not make sense to magnify ReLU' along the x-axis (it has no discernible effect), we consider a sigmoid function *f*′(*s*) = *Sigmoid*(*cs*) for some constant c. As shown in Figure 6, the performance was enhanced with c (slope at s = 0). Especially at the large limit of c (=50), the performance approached that of the original learning rules with $f^{{}^{\prime }}={f^{\prime }}_{orig}$(=ReLU') as expected.

Finally, we added normal noise as *f*′ = ${f^{\prime }}_{orig}+noise$. Figure 7 (top) shows that the learning functions fairly well as long as the noise amplitude is significantly smaller than one. The same result was observed when fixed noise was applied, as shown in Figure 7 (bottom), where the initially fixed noise was used across epochs for the same neuron. The results demonstrate robustness in learning against noise.

## 4. Discussion

Our contribution resides in demonstrating successful learning for FA_Ex-100% with a more biologically plausible connection matrix *B*. Note that *B* is a random weight matrix (vector) in the original FA paper (Lillicrap et al., 2016), whereas an all-1 vector *B* was also considered in this study. A condition on *B* for the successful learning of *W*_1_ was derived under the assumption that all activation functions are linear (i.e., linear neurons), and *W*_2_ is not trainable (related to Figure 5 in Lillicrap et al., 2016). In essence, the derived condition *W*_2_ · *B* > 0 was not satisfied before learning where *W*_2_ · *B* = 0, while *W*_2_ gets aligned with *B* during the training if *W*_2_ is also trainable. From this viewpoint, it is expected that any *B* (with a randomly initialized *W*_2_) suffices the condition *W*_2_ · *B* > 0 after the training, and FA works in the end. Thus, the condition *W*_2_ · *B* > 0 provides insight into the entire learning process, including both *W*_1_ and *W*_2_ as trainable parameters to be successful, although it does not serve as a sufficient condition for general cases rigorously. Note that what matters actually is whether the cost function (*J* = sum of squared errors) consistently decreases at each update: $\Delta J\left(W_{1},W_{2}\right)=\frac{\partial J}{{\partial W}_{1}}\cdot \Delta W_{1}+\frac{\partial J}{{\partial W}_{2}}\cdot \Delta W_{2}=-eW_{2}\cdot Be+0\;<\;0$, which leads to the condition *W*_2_ · *B* > 0 for *W*_1_ in Lillicrap et al. (2016) as the error *e* is just a scalar. Overall, there is no rigorously proven condition for successful learning, and the results in our study cannot be predicted from such a simple condition. Thus, we believe that our contribution, demonstrating successful and robust learning for a more biologically plausible *B* is not trivial.

### 4.1. What are the constraints imposed by biological plausibility?

In this study, in a narrow sense, biological plausibility means that the weight update rule can be computed only with local information that is available at a synaptic triad. Additionally, a biologically plausible learning rule should exhibit high performance comparable to that of a real brain, as the brain is unlikely to adopt a learning rule that underperforms it.

We cannot emphasize enough that rules that use only the synaptic triad are highly biologically plausible. As a variant of the FA, we derived rules that exclusively use a synaptic triad. Their performances are comparable to those of BP and FA. Among them, FA_Ex-100% is particularly elegant in the sense that its weight update rule is uniform throughout the network. This is because B = 1. The update rule is not exclusive to middle-layer neurons but also applies to output neurons. Therefore, we may only require a single learning rule for the entire brain. It is worth examining whether FA and its variants are implemented in the brain and whether the experimentally observed synaptic updates are consistent with these learning rules.

We acknowledge that FA encounters challenges as networks become deeper. Even if the FA only works with a limited number of layers, here we consider shallow networks as a model olfactory system because dopaminergic inputs are only available in a limited number of layers in the real brain. We do not claim that many trainable layers with plasticity are required to reproduce the biological brain.

It is known that animals can learn a task where a preceding cue reverses the outcomes. Therefore, it is natural to assume that the ability to solve an XOR task is necessary for an algorithm to be used in the real biological brain. We agree that this is not a sufficient condition. Although the XOR task is just a minimum-level problem that must be solved by a biological algorithm, it is ideal in that its difficulty can be controlled by changing the input dimensions to achieve a wide range of task levels. We agree that further benchmarks, possibly with larger networks, are necessary and will be left for future work.

Performing well with 3-layer ANNs is the minimum requirement. Future work should attempt more realistic network structures with untrainable layers and loops. We agree that the reality of the model can be an endless argument, although previous studies on the olfactory system have considered similar mathematical models with random connections and inputs (Hiratani and Latham, 2022). Even so, we are committed to the study of biology, and our model serves as a valuable tool in the sense that the proposed local synaptic learning rule can be easily compared with the experimental observations. We strongly believe that this new line of research involving end-to-end training under local synaptic rules will be the key to understanding human intelligence.

### 4.2. Scalability is essential for pursuing biological plausibility

In this study, tuning the task difficulty significantly changed the results. If the real tasks that the brain must solve are more difficult than the ones used here, the candidates for the learning rules should be narrowed down. Therefore, it will be essential to simulate tasks involving various difficulties in the future. For example, we use rather small noise input dimensions, d_noise_, which makes the task easy; however, in the real brain, the number of input or sensory neurons is large. However, we believe that the number of neurons in the middle layers is also large in the real brain. Therefore, the same task may be solved by increasing both numbers in a balanced manner (Hiratani and Latham, 2022). Future work may explore GPU-based simulations to increase both the input- and middle-layer neuron counts in a balanced manner.

Although we maintained a batch size of eight throughout this study, it is natural for mice to learn from each trial as an epoch or with a batch size of one. However, it is also realistic for mice to exploit the memories of several past trials. In addition, when the total sample size for training was the same, the batch size did not affect the final performance. Therefore, it is important to determine whether mice can perform tasks within a realistic number of laboratory trials (training samples). For example, Figure 2 (bottom right) shows that 100 epochs or 800 trials (with a batch size of eight) were required for training with BP or FA. This number of trials may initially appear substantial, as the brain can learn more quickly for some tasks. However, this number is primarily influenced by the task complexity and network parameters. In practice, this number can be significantly reduced by introducing more neurons in the middle layer. The brain may indeed adopt a regime of abundant middle-layer neurons. Therefore, although we set the maximum epoch to 1,000 (8,000 trials) in all figures, as it is too long for mice to perform in the laboratory experiment, this trial number can be efficiently controlled by adjusting learning parameters such as the number of middle-layer neurons.

Taken together, many issues related to biological plausibility can be rephrased as issues of scalability in the sense that the learning parameter can counterbalance task difficulty, such as input dimensions, as the performance in the large limit is unknown. Checking the scalability requires future work, and it is necessary to elucidate, possibly with GPUs, the types of regimes used by the brain.

We agree that high-dimensional experiments using larger networks assisted by GPUs are desirable. However, not only GPU availability but also software development is pivotal for scaling; the existing frameworks for deep learning are mostly prepared for the backpropagation learning rule. Therefore, implementing a handmade learning rule without explicit cost functions for training is challenging. Although we are currently developing original Python code to utilize GPUs for handmade learning rules, we believe that this is worth further work. We aim to publish the current paper separately using highly readable code specialized for attached CPUs.

### 4.3. From spiking models to rate-based models

In this study, we focused exclusively on rate-based learning rules and did not discuss spike timing. However, it is easy to bridge the gap between these two approaches by starting with small time bins and subsequently averaging them, which results in rate-based learning rules. This is because FA variants require only the spike frequencies of presynaptic, postsynaptic, and dopaminergic neurons.

## Data availability statement

The original contributions presented in the study are included in the article/supplementary material, further inquiries can be directed to the corresponding author.

## Author contributions

MK performed the numerical simulations. KI contributed to the discussion on biological plausibility. KM wrote the manuscript. All authors contributed to the manuscript revision and have read and approved the submitted version.
